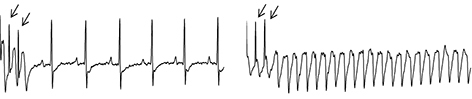# Treatments for cardiac arrhythmias in Rett syndrome

**Published:** 2015-04

Rett syndrome (RTT) is a neurological disorder in which sudden cardiac death is common. Cardiac arrhythmias – which are frequent in this disease – are thought to contribute to sudden death. Individuals with RTT often have long QT (LQT), an abnormal prolongation of the QT interval in the heart cycle. Na^+^-channel- and β-blockers (such as phenytoin and propanolol, respectively) are being tested for LQT treatment in animal models, but further studies are needed to fully characterise their action, in particular when administered chronically. To investigate this, Jeffrey L. Neul and collaborators tested the effects of chronic treatment with either propanolol or phenytoin in RTT mice (which bear mutations in the RTT-related gene methyl-CpG-binding protein 2). They found that phenytoin but not propranolol normalises the LQT arrhythmia. In addition, phenytoin ameliorated the obesity phenotype often associated with RTT. However, it surprisingly worsened the breathing patterns in male mice. Notably, Na^+^-channel blockers ameliorated the LQT anomaly in individuals with RTT. The study suggests that Na^+^-channel blockers could be a treatment option for RTT-affected individuals with LQT. Further animal studies are however needed to disclose LQT causes and develop more selective drugs. **Page 363**

**Figure:**